# On conductance-based neural field models

**DOI:** 10.3389/fncom.2013.00158

**Published:** 2013-11-12

**Authors:** Dimitris A. Pinotsis, Marco Leite, Karl J. Friston

**Affiliations:** The Wellcome Trust Centre for Neuroimaging, University College LondonLondon, UK

**Keywords:** neural field theory, mean field modeling, electrophysiology, conductance based models, dynamic causal modeling

## Abstract

This technical note introduces a conductance-based neural field model that combines biologically realistic synaptic dynamics—based on transmembrane currents—with neural field equations, describing the propagation of spikes over the cortical surface. This model allows for fairly realistic inter-and intra-laminar intrinsic connections that underlie spatiotemporal neuronal dynamics. We focus on the response functions of expected neuronal states (such as depolarization) that generate observed electrophysiological signals (like LFP recordings and EEG). These response functions characterize the model's transfer functions and implicit spectral responses to (uncorrelated) input. Our main finding is that both the evoked responses (impulse response functions) and induced responses (transfer functions) show qualitative differences depending upon whether one uses a neural mass or field model. Furthermore, there are differences between the equivalent convolution and conductance models. Overall, all models reproduce a characteristic increase in frequency, when inhibition was increased by increasing the rate constants of inhibitory populations. However, convolution and conductance-based models showed qualitatively different changes in power, with convolution models showing decreases with increasing inhibition, while conductance models show the opposite effect. These differences suggest that conductance based field models may be important in empirical studies of cortical gain control or pharmacological manipulations.

## Introduction

This paper introduces a conductance-based neural field model that accounts for spatial variations in synaptic transmission among neural ensembles on the cortical surface. Our modeling draws from computational neuroscience, in which spiking models are described by population density dynamics. Generally, in these mean field approaches, population activity is expressed in terms of mean post-synaptic voltages and currents; however, conductance based models that consider the geometry and topography of neuronal interactions are relatively rare in the literature (Goldstein and Rall, 1974; Ellias and Grossberg, 1975; Somers et al., 1995; Ermentrout, 1998); in other words, the spatiotemporal dynamics of conductance models are often simplified to neural mass approximations, such that the population density depends upon time only. In our model, we make the statistics of neuronal states a function of space, thereby characterizing mean spike rates as fluctuations propagating over horizontal cortical connections. This involves using wave equations to describe interactions between spatially extended neuronal populations, in terms of changes in the flow of post-synaptic currents, the history of pre-synaptic inputs and the action of certain neuromodulators.

Conductance-based models have a long history in mathematical neuroscience; for a detailed review, see (Tuckwell, 2005). Within the setting of dynamic causal modeling, a treatment of conductance-based models (that ignores the spatial distribution of sources over the cortex) can be found in (Marreiros et al., 2010) that was later applied to characterize synaptic function empirically (Moran et al., 2011b). These models regard a neuron as an electrical circuit, where the membrane response follows the inflow or outflow of current through ionic channels. These channels are associated with conductances that depend upon electrochemical gradients across the membrane and the configuration of various ion channels and receptors. The standard kinetic model for *conductance* dynamics comprises two sorts of equations: (1) an equation for the rate of change of transmembrane potential as an aggregate current flux—consisting of Ohmic components and (2) equations for the channel conductances that depend upon pre-synaptic spiking and the proportion of open channels. This form of modeling necessarily entails non-linear terms, in which changes in post-synaptic potential involve the product of synaptic conductances and potential differences associated with different channel types. In other words, the equations of motion for neuronal states are necessarily non-linear and second-order (with respect to the hidden neuronal states), in accord with electromagnetic laws. This should be contrasted with the alternative approach to neural mass and mean field modeling based upon *convolution* operators. In these models, post-synaptic depolarization is modeled as a (generally linear) convolution of pre-synaptic spiking input. Crucially, this convolution can be formulated in terms of linear differential equations.

In short, the key distinction between conductance and convolution based models is that conductance based models have non-linear dynamics and, in principle, provide a degree of biological realism that can incorporate neuromodulatory and other conductance-specific physiological effects. Here, we use this basic form of model to describe the depolarization and conductances of neural fields on the cortical sheet—and recast pre-synaptic spike rates as fluctuations obeying a wave equation that propagates along axon collaterals. We adopt a neural mass approach, where the input to each neuron of the population is the expected firing over all neurons around a point on a local cortical surface or patch. We thus obtain a conductance-based cortical field model linking population dynamics to synaptic neurotransmission. This paper focuses on the operational aspects of this model and its ability to reproduce typical cortical responses such as event–related potentials (ERPs) and cross-spectral densities.

The use of conductance based models to simulate large networks of neurons has enjoyed recent developments, involving both direct simulations of large numbers of neurons (which can be computationally expensive); e.g., (Izhikevich, 2004) and probabilistic approaches; e.g., (De Groff et al., 1993; Nykamp and Tranchina, 2000). Probabilistic approaches model the population density directly and bypass direct simulations of individual neurons. We follow a similar approach that exploits a neural mass approximation. This effectively replaces coupled Fokker-Planck equations describing population density dynamics, with equations of motion for expected neuronal states; that is, their first moments. These equations are formulated in terms of the mean of the population density over each neuronal state, as a function of space.

Recent work has considered the link between networks of stochastic neurons and neural field theory by using convolution models (with alpha type kernels) to characterize post-synaptic filtering: some studies have focused on the role of higher order correlations, starting from neural networks and obtaining neural field equations in a rigorous manner; e.g., (Buice et al., 2010; Touboul and Ermentrout, 2011), while others have considered a chain of individual fast spiking neurons (Rose and Hindmarsh, 1989), communicating through *spike* fields (Wilson et al., 2012). These authors focused on the complementary nature of spiking and neural field models and on eliminating the need to track individual spikes (Robinson and Kim, 2012). Our focus is on the behavior of neuronal populations, where conductance dynamics replace the convolution dynamics—and the input *rate* field is a function of both time and space. This allows us to integrate field models to pre-dict responses and therefore, in principle, use these models as generative or observation models of empirical data.

When modeling pre-synaptic firing rate, we use the approximation of (Robinson et al., 1997) that yields broad temporal pulses in response to a delta input. Crucially, we characterize the neuronal input as fluctuating mean spiking activity that conforms to a wave equation. Our model is non-linear in the neuronal states, as with single unit conductance models and the model of (Liley et al., 2002). This model entails a multiplicative non-linearity, involving membrane depolarization and pre-synaptic input and has successfully reproduced the known actions of anaesthetic agents on EEG spectra, see e.g., (Steyn-Ross et al., 2001, 2011; Liley et al., 2003; Bojak and Liley, 2005; Wilson et al., 2006). Our model is distinguished by the fact that it incorporates distinct cell types with different sets of conductances and local conduction effects. More specifically, it comprises three biologically plausible populations, each endowed with excitatory and inhibitory receptors. It focuses on the propagation of spike rate fluctuations over cortical patches and the effect this spatiotemporal dynamics has on membrane dynamics gated by ionotropic receptor proteins. We consider laminar specific connections among two-dimensional populations (layers) that conform to canonical cortical microcircuitry. The parameterization of each population or layer involves a receptor complement based on findings in cellular neuroscience. However, this model incorporates lateral propagation of neuronal spiking activity that is parameterized through an intrinsic (local) conduction velocity.

This note comprises three sections. In the first, we review the mathematical formalism that underlies conductance based neural field models and introduce a generative model that accounts for both conductance effects on membrane dynamics and propagation of activity along intrinsic connections. In the second, we compare the behavior of this model with the corresponding behavior of convolution field models and consider the effect of changing model parameters. We also compare and contrast responses obtained by the neural mass reductions of these (conductance and convolution) models; in other words, models that consider dynamics over time only. Our focus here is on the effect that propagating fluctuations along horizontal (intrinsic) connections have on spatiotemporal dynamics. We conclude with a discussion of how the neural field model based upon first-order statistics—used in this paper—relates to formal treatments of population dynamics.

## A conductance-based neural field model

We consider a group of *N*_*R*_ interacting neuronal populations or layers. The collective dynamics (activity) of each population evolve according to a set of coupled differential equations that depend on some scalar quantities or neuronal states *q*(*x*, *t*) ∈ {*v*(*x*, *t*), *g*_*k*_(*x*, *t*), μ_*k*_(*x*, *t*)} that are continuous functions of the location on the cortical surface *x* ∈ *X*. These neuronal states include the transmembrane potential *v*(*x*, *t*), a set of synaptic conductances *g*_*k*_(*x*, *t*) modeling distinct membrane channel types and the pre-synaptic input to which they are exposed μ_*k*_(*x*, *t*).

The resulting populations can be viewed as a set of coupled RC circuits, where channels open in proportion to pre-synaptic input and close in proportion to the number already open. Changes in conductance produce changes in depolarization in proportion to the potential difference between transmembrane potential and a reversal potential *v*_*k*_ that depends upon the channel type. Open channels result in hyperpolarizing or depolarizing currents depending on whether the transmembrane potential is above or below the reversal potential. These currents are supplemented with exogenous current *u*(*x*, *t*) to produce changes in the transmembrane potential (scaled by the membrane capacitance *C*). The first order moments or means of neuronal states at a location *x* on a cortical patch evolve according to the following system of differential equations:

(1) $$\begin{array}{l} C\dot{v}(x,t)=\sum_{k}g_{k}\left(v_{k}-v(x,t)\right)\;\\ {\dot{g}}_{k}(x,t)=\lambda_{k}\left(\mu_{k}(x,t)-g_{k}(x,t)\right)\\ \mu_{k}(x,t)=\iint d\left(x-x^{\prime },t-t^{\prime }\right)\sigma_{k}\left(v\left(x^{\prime },t^{\prime }\right)\right)\text{​}dt^{\prime }dx^{\prime }+u(x,t) \end{array}$$

where pre-synaptic input to point *x* arises from a spatiotemporal convolution of a sigmoid activation function of depolarizations in other locations *x*' (in the past at time *t*') and *k* = *E*, *I* denote excitatory and inhibitory synaptic conductances or inputs. This model assumes that each neuron senses all others, so that endogenous input is the expected firing of contributing locations summarized with a sigmoid function σ_*k*_(v) of their transmembrane potential. It is this function that accommodates the stochastic dispersion of neuronal states: see (Marreiros et al., 2010) for a detailed discussion. The rate constants λ_*k*_ characterize the response of each channel type to afferent input. Finally, *d*(*x*, *t*) is a connectivity kernel that accommodates axonal propagation delays. It is this connectivity kernel that specifies the spatial aspects of the ensuing spatiotemporal dynamics.

A ubiquitous choice for the connectivity kernel (Wilson and Cowan, 1973; Jirsa and Haken, 1996) is based on the assumption that the number of synaptic connections between populations decays exponentially with some characteristic spatial scale *c*; namely, *d*(*x*, *t*) = *ae*^−*c*· |*x*|^ δ(t−|*x*|/*s*), where *a* scales connection strengths and *s* is the speed at which neuronal spikes propagate down connections. This assumption means that we can express the dynamics of the mean firing rates as (see e.g., Pinotsis et al., 2012):

(2) $$\begin{array}{l} {\ddot{\mu }}_{k}(x,t)+2sc{\dot{\mu }}_{k}(x,t)-s^{2}\left(\partial_{xx}\mu_{k}(x,t)-c^{2}\mu_{k}(x,t)\right)\;\\ \;=as^{2}c\sigma \left(v(x,t)\right)+u \end{array}$$

Combining Equations (1) and (2) gives us the equations of motion for all neuronal states:

(3) $$\dot{q}(x,t)=\left[\begin{array}{c} \dot{v}\\ {\dot{g}}_{k}\\ {\dot{\mu }}_{k}\\ {{\dot{\mu }}^{\prime }}_{k} \end{array}\right]=\left[\begin{array}{c} \frac{1}{C}\sum_{k}g_{k}\left(v_{k}-v(x,t)\right)\\ \lambda_{k}\left(\mu_{k}(x,t)-g_{k}(x,t)\right)\\ {\mu^{\prime }}_{k}(x,t)\\ -2sc{\mu^{\prime }}_{k}(x,t)+s^{2}(\partial_{xx}\mu_{k}(x,t)\\ -c^{2}\mu_{k}(x,t))+as^{2}c\sigma (v(x,t))+u \end{array}\right]$$

for the quantitative purposes of this paper, we solve Equation (3) using a simple finite differences scheme for the second-order spatial derivatives.

**Figure 1:**
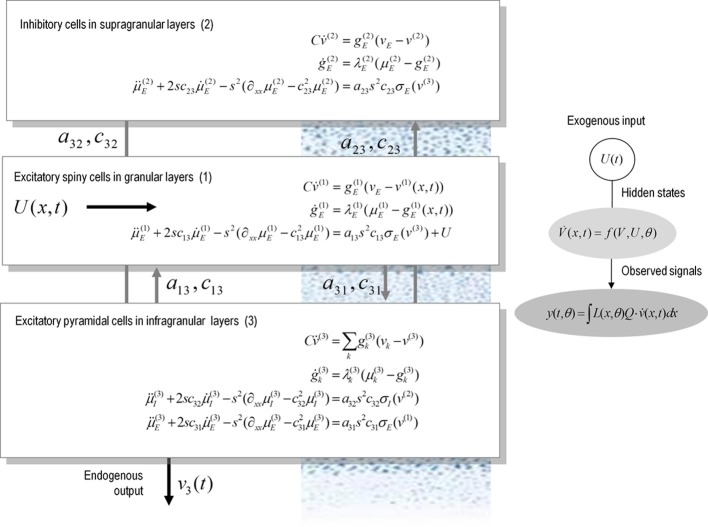
**A conductance-based neural field model.** This schematic summarizes the equations of motion or state equations that specify a conductance based neural field model of a single source. This model contains three populations, each associated with a specific cortical layer. These equations describe changes in expected neuronal states (e.g., voltage or depolarization) that subtend observed local field potentials or EEG signals. These changes occur as a result of propagating pre-synaptic input through synaptic dynamics. Mean firing rates within each layer are then transformed through a non-linear (sigmoid) voltage-firing rate function to provide (pre-synaptic) inputs to other populations. These inputs are weighted by connection strengths and are gated by the states of synaptic ion channels.

Figure 1 illustrates the model for a spatially extended cortical source, which we will call a conductance-based neural field. In this model, the source consists of three layered populations; namely, spiny stellate cells, inhibitory interneurons and pyramidal cells. Each population is assigned to a cortical layer and is connected to other layers according to the principles of a typical cortical microcircuit (as described in e.g., Pinotsis et al., 2012). Each layer is equipped with neural states *q*(*x*, *t*) ∈ {*v*^(*i*)^(*x*, *t*), *g*^(*i*)^_*k*_(*x*, *t*), μ^(*i*)^_*k*_(*x*, *t*), μ^′(*i*)^_*k*_(*x*, *t*)}, where the superscript *i* indexes different laminar populations—and the states evolve according to a system of coupled equations of the form of Equation (3). When this model is augmented with a mapping from source to sensor space, we obtain a generative model of electrophysiological responses that can be used to infer the parameters of both synaptic kinetics—and intrinsic or lateral interactions, through the parameters of the connectivity kernel. Crucially, because of the biologically realistic construction of this model, one can examine the dependency of spatially extended dynamics of particular conductances and receptor subtypes.

## Relation to classical neural field models

Equation (3) is an equation of motion, describing a neuronal field in terms of expected neuronal states. This sort of equation can accommodate both convolution and conductance based neural field models. Convolution neural field models involve kernels that are linear in the states; for example *q*(*x*, *t*) ∈ {*v*(*x*, *t*), *v*′(*x*, *t*), μ(*x*, *t*), μ′(*x*, *t*)}. These models can also be cast in a form similar to Equation (3):

(4a) $$\dot{q}(x,t)=\left[\begin{array}{c} \dot{v}\\ {\dot{v}}^{\prime }\\ \dot{\mu }\\ {\dot{\mu }}^{\prime } \end{array}\right]=\left[\begin{array}{c} v^{\prime }\\ -2\lambda v^{\prime }(x,t)-\lambda^{2}v(x,t)\\ \;+H\lambda \mu (x,t)\\ \mu^{\prime }(x,t)\\ -2sc\mu^{\prime }(x,t)+s^{2}\text{​}(\partial_{xx}\mu (x,t)\\ -c^{2}\mu (x,t))+as^{2}c\sigma (v(x,t))+u \end{array}\right]$$

Indeed, this equation can be rewritten as

(4b) $$\begin{array}{l} \;\ddot{v}(x,t)+2\lambda v^{\prime }(x,t)+\lambda^{2}v(x,t)=H\lambda \mu (x,t)\\ \ddot{\mu }(x,t)+2sc\dot{\mu }(x,t)-s^{2}(\partial_{xx}\mu (x,t)-c^{2}\mu (x,t))=as^{2}c\sigma (v(x,t))+u \end{array}$$

These equations describe neural fields with constant coefficients in homogeneous media; see e.g., Pinotsis and Friston, 2011; Pinotsis et al., 2012, 2013. In a previous paper, we introduced a neural field model involving the three laminar populations depicted in Figure 1, which we called a Jansen and Rit neural field model. This model is similar to the classical Wilson and Cowan or Amari models (Wilson and Cowan, 1972; Amari, 1977). The model in Equation (4) assumes an alpha-type synaptic convolution kernel. This is simply the Green's function associated with a linear filtering of pre-synaptic input to produce changes in depolarization. In these mean field models, passive membrane dynamics and dendritic effects are summarized by lumped parameters (λ and *H* in the above equations) that model the rate that depolarization increases to a maximum and synaptic efficacy (or maximum post-synaptic potential), respectively. However, this sort of description neglects the timescales of synaptic currents that are implicit in conductance based models: in Equations (3) these timescales are characterized in terms of the rate constants λ and *C*; namely, channel response and membrane capacitance.

The crucial difference between these (linear and non-linear) parameterizations is that in the conductance models, the parameters characterize the response of each population to distinct excitatory and inhibitory inputs: in other words, there is a set of synaptic rate constants (each corresponding to a distinct channel) associated with each population. The corresponding dynamics are defined over timescales that result from the parameters used and the non-linear interaction between membrane potential and conductance. These timescales may be crucial in pharmacological manipulations that selectively affect one sort of current in a receptor specific fashion. This means that conductance-based models may be more appropriate candidates to study synaptic function at the level of specific neurotransmitter systems (Faulkner et al., 2009; Moran et al., 2011a).

## Simulations

In the following, we focus on simulated responses generated by convolution and conductance variants of neural mass and field models—where these two variants incorporate fundamentally different descriptions of post-synaptic filtering. We investigate the dependence of simulated responses on model parameters with neurobiological or pharmacological significance. Specifically, we examine: (1) the effects of changing synaptic parameters and (2) the qualitative differences in the behavior of convolution and conductance based models. In this technical note, we focus only on the phenomenology of the models in the domains of the parameter space that are dynamically stable.

We generated synthetic electrophysiological responses by integrating equations (3) or (4) from their fixed points and characterized the responses to external (excitatory) impulses to spiny stellate cells, in the time and frequency domain. The spectral responses correspond to the model's transfer function. Electrophysiological signals (LFP or M/EEG data) were simulated by passing neuronal responses through a lead field that varies with location on the cortical patch. The resulting responses in sensor space (see Figures 5–7) are given by a mixture of currents flowing in and out of pyramidal cells in Figure 1:

(5) $$y(t,\theta )=\int L(x,\theta )Q\cdot \dot{v}(x,t)dx$$

In this equation, *Q*⊂ θ is a vector of coefficients that weight the relative contributions of different populations to the observed signal and *L*(x, θ) is the lead field. This depends upon parameters θ and we assume it is a Gaussian function of location—as in previous models of LFP or MEG recordings: see (Pinotsis et al., 2012). This equation is analogous to the usual (electromagnetic) gain matrix for equivalent current dipoles. We assume here that these dipoles are created by pyramidal cells whose current is the primary source of an LFP signal. With spatially extended sources (patches), this equation integrates out the dependence on the source locations within a patch and provides a time series for each sensor.

We modeled a cortical source (approximated with 11 grid points) and used the model equations (see Figure 1) to generate evoked responses (impulse response functions) and associated transfer functions (their Fourier transform). The parameters of this model are provided in Table 1. The results reported below were chosen to illustrate key behaviors in terms of ERP (impulse response) and frequency responses (transfer functions) in sensor space, following changes in parameter values. We compare and contrast results from the two classes of models (conductance and convolution models). We also consider the corresponding result for their mass variants, which use the same equations but assume that all neurons of a population are located at (approximately) the same point.

**Table 1:** **Parameters of conductance-based neural field and mass models**.

**Parameter**	**Physiological interpretation**	**Value**
*g*_*L*_	Leakage conductance	1
α_13_, α_23_, α_31_, α_32_	Amplitude of intrinsic connectivity kernels	(1/10, 1, 1/2, 1)*3/10 (field) 1/2, 1, 1/2, 1 (mass)
*c*_*ij*_	Intrinsic connectivity decay constant	1 (mm^−1^)
*v*_*L*_, *v*_*E*_, *v*_*I*_	Reversal potential	−70, 60, −90 (mV)
*v*_*R*_	Threshold potential	−40 (mV)
*C*	Membrane capacitance	8 (pFnS^−1^)
s	Conduction speed	0.3 m/s
λ, λ˜	Post-synaptic rate constants	1/4, 1/16 (ms^−1^)
ℓ	Radius of cortical patch	7 (mm)

The resulting mass models include the well-known Jansen and Rit mass model, see (David and Friston, 2003) and the simplified Morris-Lecar type model (that neglects fast voltage-dependent conductances) introduced in (Marreiros et al., 2010). This conductance-based model is based on the Rall and Goldestein equations (Goldstein and Rall, 1974) and is formally related to Ermentrout's (Ermentrout, 1998) reduction of the (Somers et al., 1995) model. Mass models have often been used to characterize pharmacological manipulations and the action of sedative agents (Traub et al., 1999; Liley et al., 2003; Bojak and Liley, 2005; Moran et al., 2008; Hutt and Longtin, 2010; Steyn-Ross et al., 2011). This usually entails assuming that a neurotransmitter manipulation changes a particular parameter, whose effects are quantified using a contribution or structural stability analysis, where structural stability refers to how much the system changes with perturbations to the parameters.

Our aim here was to illustrate changes in responses with changes in the parameters of the convolution and conductance field models. A range of anaesthetics has been shown to increase inhibitory neurotransmission. This effect has been attributed to allosteric activators that sensitize GABA_A_ receptors. In the context of our models, these effects correspond to an increase of the strength of inhibitory input to pyramidal cells *a*_32_. We here focus on spectral responses in the alpha and beta range, as this is the range of interest for many applications involving drug effects.

**Figure 2:**
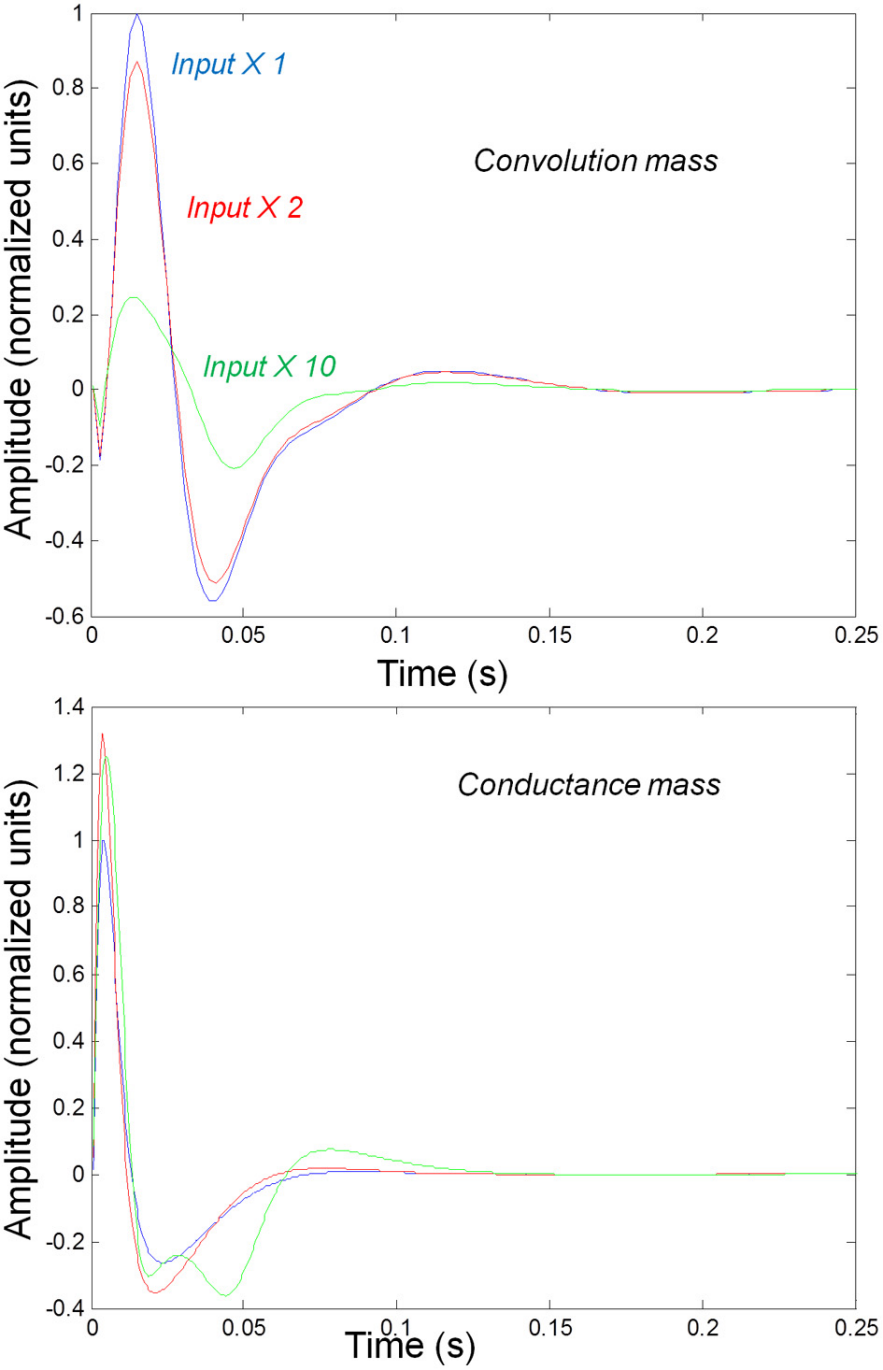
**Responses to impulses of different amplitudes for convolution (top) and conductance (bottom) based neural mass models.** The responses are normalized with respect to the amplitude of each input. The blue lines illustrate responses to small perturbations. The red lines illustrate responses to intermediate sized inputs, where conductance based models show an augmented response, due to their non-linearity. The green lines show responses for larger inputs, where the saturation effects due to the sigmoid activation function are evident.

We first consider generic differences in non-linear processes mediated by conductance and convolution based models. To do this, we integrated the corresponding equations for (impulse) inputs of different amplitudes and plotted temporal responses resulting from fixed point perturbations. Linear models are insensitive to the amplitude of the input, in the sense that the impulse responses scale linearly with amplitude. Our interest here was in departures from linearity—such as saturation—that belie the non-linear aspects of the models. Figure 2 shows the responses of the mass models to an impulse delivered to stellate cells. Note that these responses have been renormalized with respect to the amplitude of each input. The red (green) curves depict responses to double (ten times) the input reported by the blue curves. We used the same parameters for both models: see Table 1; where additional parameters for the Jansen and Rit model are provided in Table 2 below.

**Table 2:** **Parameters of convolution-based neural field and mass models**.

**Parameter**	**Physiological interpretation**	**Prior mean**
*H*_E_, *H*_I_	Maximum post-synaptic depolarizations	8 (mV)
α_13_, α_23_, α_31_, α_32_	Amplitude of intrinsic connectivity kernels	(1/2, 1, 1/2, 1)*3/10 (field) 1, 4/5, 1/4, 1 (mass)

Other parameters as in **Table 1**.

It can be seen that there are marked differences between the model responses. The top panel depicts the response of the convolution mass model and the lower panel shows the equivalent results for the conductance model. One can see that large inputs produce substantial sub-additive saturation effects (blue vs. green lines in Figure 2): for the convolution model, increasing the input amplitude produces a sub additive increase in response amplitude; whereas for the conductance model, the non-linearities produce an inverted U relationship between the amplitude of the response, relative to the input. In summary, the form of the input-output amplitude relationship differs quantitatively for the conductance (inverted U) and convolution (decreasing) models (see Figure 2).

**Figure 3:**
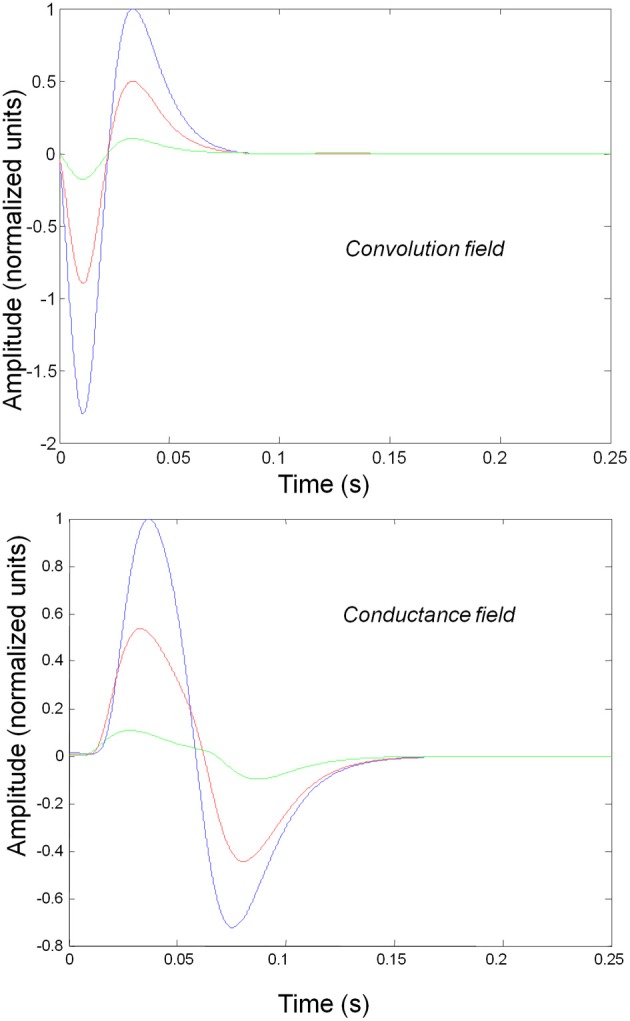
**Impulse response of conductance and convolution field models to inputs of various amplitudes distinguished by different colours as in Figure 2.** The system's flow is generated by Equations (3) and (4a) and the model parameters are given in Tables 1, 2. Non-linear effects are more pronounced—with attenuation of the response amplitude, even for intermediate input amplitudes.

Figure 3 shows the impulse responses of the field models described by Equations (3) and (4). Here we observe sub-additive saturation effects that are similar to the responses of the convolution mass model—with relatively stronger attenuation of the response amplitude than the mass model even for intermediate input amplitudes.

We next characterized the spectral responses of convolution and conductance-based neural fields and their mass variants. It should be noted that this analysis is purely phenomenological and a complete bifurcation analysis will be presented elsewhere. Here, we focus on transfer functions associated with the models. These are shown in subsequent figures for a range of physiological parameters. The transfer functions can be regarded as the spectral density that would be seen if the field and mass models were driven by independent fluctuations. It is interesting that—for the biologically plausible parameter values we use—both field and mass models exhibit alpha peaks (as opposed to a *1/f* scale invariant form) that are typical of neural field models (Nunez, 1995; Robinson et al., 2001; Liley et al., 2002). Note that the transfer function characterizations used below assume a linearization around the fixed point and therefore do not capture the non-linear behavior of the models.

**Figure 4:**
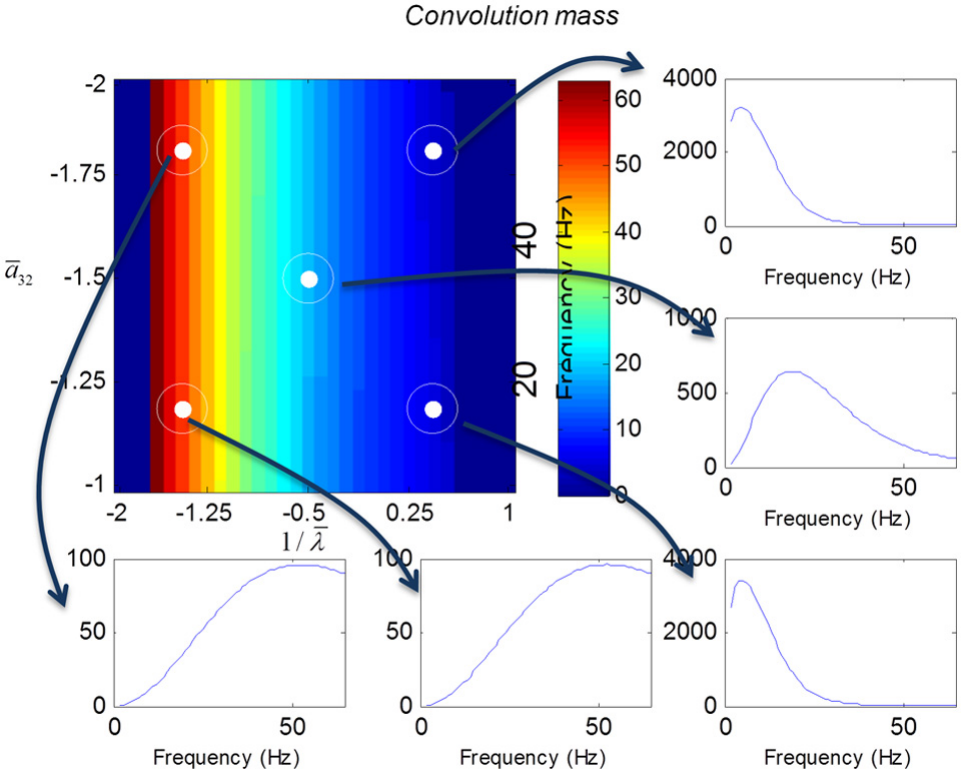
**Transfer functions associated with a convolution mass model when changing the excitatory time constant and the connection driving the pyramidal cells over a log-scaling range of (−2, 1) *x* (−2, −1) (from top to bottom and left to right).** The image format summarizes the transfer function in terms of its peak frequency. Transfer functions can be regarded as the spectral response that would be seen if the model was driven by independent (white) fluctuations. They are also the Fourier transform of the impulse response functions of the previous figures.

**Figure 5:**
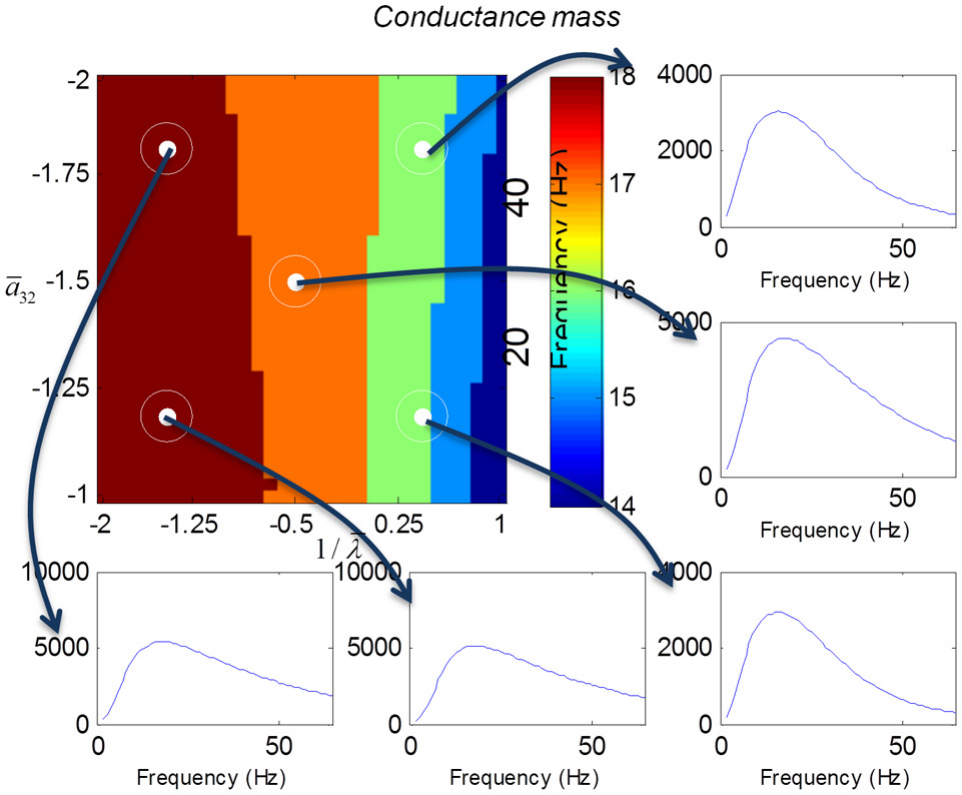
**This figure shows the transfer functions of a cortical source described by a conductance mass model.** Here, the intrinsic connectivity and excitatory time constant are changed as in Figure 4. Note the alpha and beta peaks that are typical of these models.

We varied the inhibitory intrinsic connectivity, *a*_32_ and excitatory time constant, 1/λ, of the inhibitory populations between 10 and 36% and between 10 and 270%, respectively, of the values in Tables 1, 2 (this corresponds to a log-scaling of between minus two and minus one and minus one and plus one, respectively). We denote these new values by ā_32_ and 1/λ, respectively. The transfer functions for the neural mass variants of the convolution and conductance models are shown in Figures 4, 5, respectively. The images in subsequent figures report the peak frequency of the spectral and response as a function of the two model parameters (the peak frequency corresponds to maximum system response). Exemplar transfer functions for selected parameter value pairs are shown as functions of frequency. We focus on spectral responses produced by fixed point perturbations; where lack of convergence to a fixed point is encoded by dark blue regions in the images.

**Figure 6:**
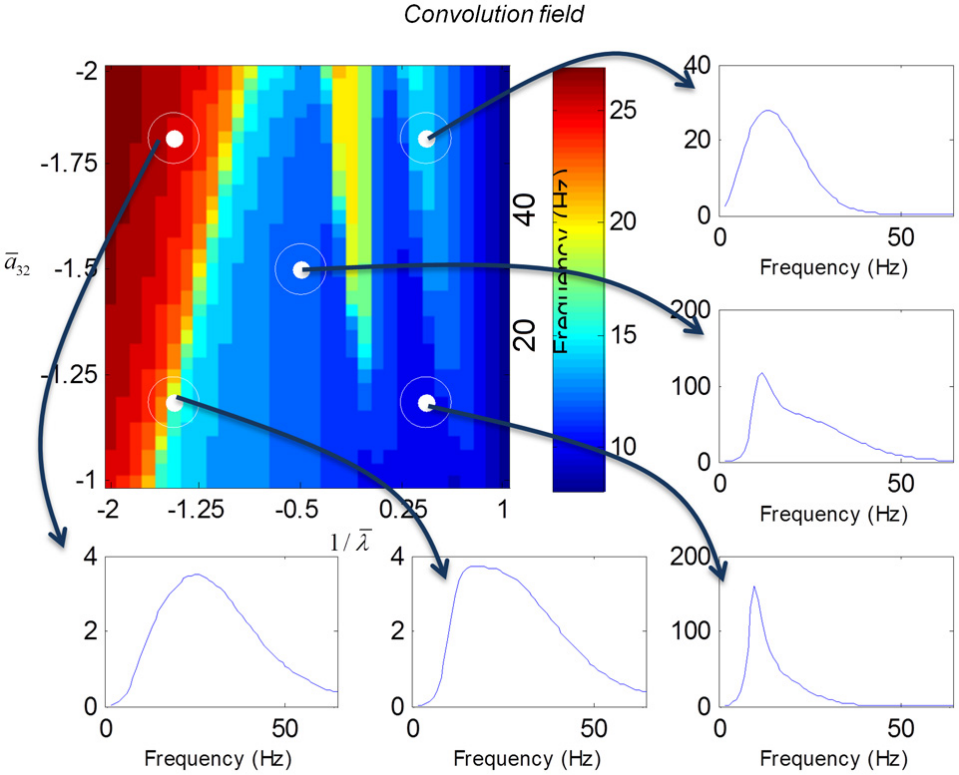
**Transfer functions associated with a convolution field model.** These are equivalent to the transfer functions shown in Figure 4, where we now model spatial propagation effects with a wave equation. Here, one observes the characteristic increase in frequency when the time constants decrease.

**Figure 7:**
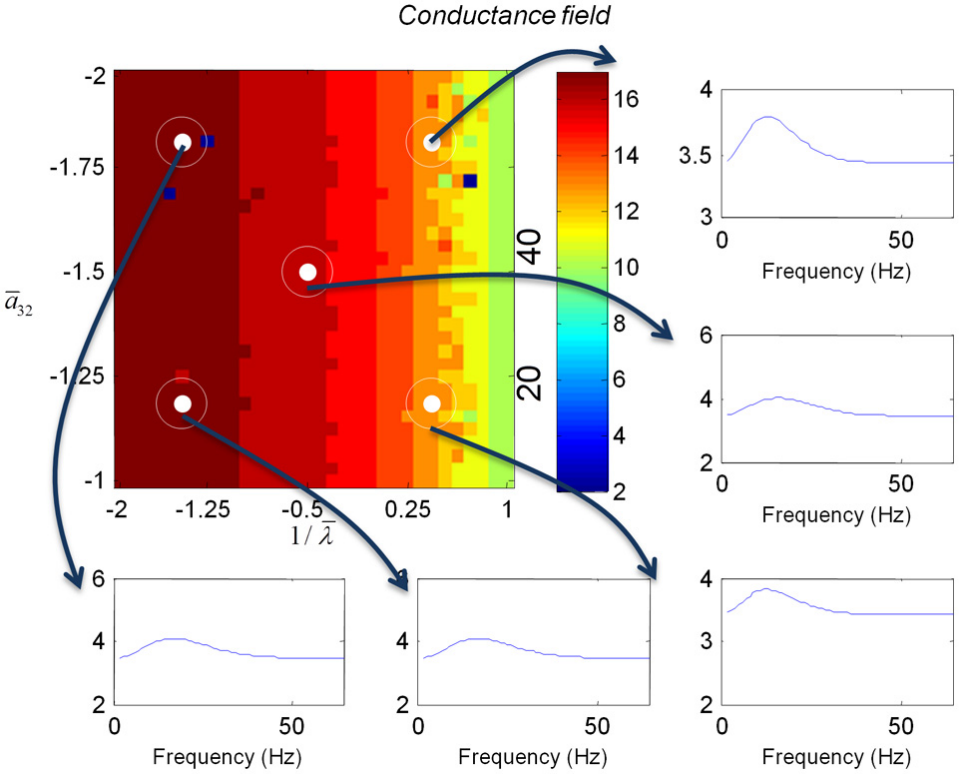
**This figure shows the changes in the transfer function of a conductance field model.** This is the equivalent to the results for the mass model in Figure 5, where we now include spatial propagation effects.

In mass models, the peak frequencies of the spectra reflect the alpha and beta activity that these models are known to produce. It is interesting that the most parsimonious among all models considered (the convolution mass model) seems to support the widest range of simulated peak frequencies; this is, however, not a conclusive result as it is heavily dependent on the particular parameterization chosen—a fuller exploration of the parameter space will be the focus of future work. A common pattern observed in all models is an increase of peak frequencies with smaller time constants of the inhibitory populations. In other words, as the strength of inhibition increases, activity becomes progressively faster (power shifts to higher frequencies). Conversely, convolution and conductance mass models showed quantitatively different changes in power, with convolution models showing decreases with increasing inhibition, while conductance models show the opposite effect. The transfer functions for the corresponding field models are shown in Figures 6, 7. Here, one observes that responses of the convolution model are similar to those obtained from the mass models above—dominated by changes in the rate λ parameter with less sensitivity to changes in the connectivity parameter. Again, we see a common increase in frequency as the inhibitory rate parameter is increased (or the time constant is decreased)—and the opposite effects under convolution and conductance models, in terms of power.

**Figure 8:**
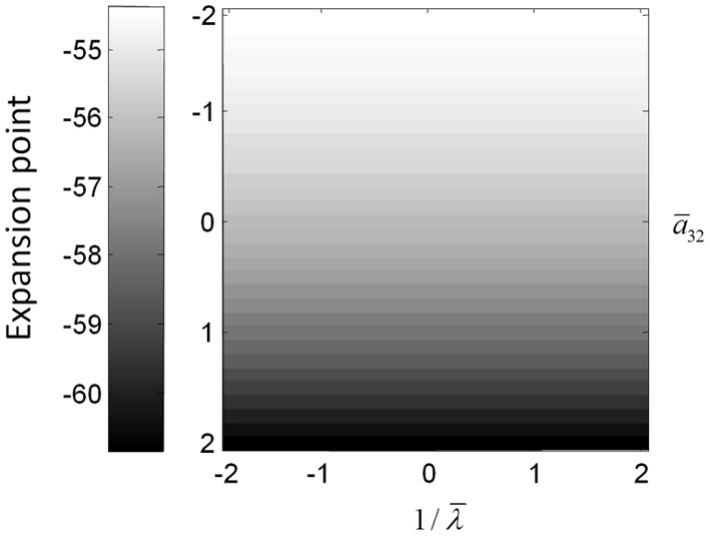
**(Top) Mean depolarization of the pyramidal population of the conductance neural mass model as a function of parameter changes.** This corresponds to the fixed point around which the transfer functions in Figure 5 were computed.

The above illustrations of system's predictions assume that spectral responses result from fixed point perturbations. For conductance models, a change in the parameters changes both the expansion point and the system's flow (provided the flow is non-zero). Figure 8 shows the dependence of the conductance model's fixed points on parameter perturbations. The model parameterization used here renders the expansion point relatively insensitive to changes in the synaptic time constant. Figure 8 shows the results for the conductance mass model; results for its field variant were very similar.

## Discussion

In this paper, we have introduced a conductance based neural field model that combines biologically realistic synaptic dynamics—based explicitly on transmembrane currents—with neural field equations, describing the propagation of spikes over the cortical surface. This model allows for fairly realistic inter- and intra-laminar intrinsic connections over a spatially extended cortical surface that give rise to neuronal dynamics. We have focused on the time evolution of expected neuronal states that underlie observed electrophysiological signals (such as LFP recordings and EEG). This time evolution characterizes the model's transfer functions and implicit spectral responses to uncorrelated input. Our main finding is that both the evoked responses (impulse response functions) and induced responses (transfer functions) show quantitative differences depending upon whether one uses a neural mass or field model. It is interesting that field models do not always produce a wider range of spectral responses for equivalent changes in their parameters (despite their greater degrees of freedom, compare Figures 4, 6). Similarly, conductance field models do not necessarily show a greater sensitivity to small parameter perturbations in comparison with their convolution counterparts (that appear more parsimonious). Although, overall, all models reproduce the characteristic increase in frequency when the rate constants of inhibitory populations increase, the precise frequency dependency depends sensitively on model type. The choice of the appropriate model might therefore depend on the particular research question at hand: for example, whether the focus is on topographic as opposed to intrinsic neurotransmitter properties or drug effects etc. This choice may also be informed by previous applications, where similar models have already proven useful along with the particular modality considered (see also the discussion in Pinotsis et al., 2013). Conductance field models may be useful in applications such as dynamic causal modeling, that try to quantify changes in gain control in cortical circuits or explain pharmacological manipulations.

The models considered in this paper deal only with the expected values (means) of neuronal states. This contrasts with higher order field treatments that would consider not just fluctuations in the means or first-order statistics of population dynamics but also higher-order statistics—such as the covariance among different neuronal states within a population or ensemble. In principle, it is relatively easy to extend the formalism described in this paper to cover the dynamics of both means and covariances using the Laplace approximation (a.k.a. the method of moments). In these generalizations, one considers the distribution over the neuronal states of a given population to have a Gaussian form 𝒩(*q*(*x*, *t*), Σ(*x*, *t*)). Crucially, the equations of motion now pertain to both the expectations and the covariances (Marreiros et al., 2010). The interesting challenge for the neural field variants of these Laplace models is that the covariances have a spatial dimension and, essentially, become spatial covariance functions (cf., Gaussian processes or random fields). The implicit covariance functions of space have a smoothness that is determined by the intrinsic connectivity kernels and the dynamics of the first order statistics.

These equations of motion for the means and covariances reduce to the neural fields considered in this paper when the off-diagonal terms of the covariance matrix Σ (*x*, *t*) are zero. In this special case, the dynamics of the means and covariances are uncoupled and one can assume a fixed covariance (as in Equations 3 and 4): see Marreiros et al. (2010) for details. More generally, full mean field treatments can provide higher order corrections to *stochastic* neural field models and offer an alternative description of the motion of their sufficient statistics, cf., (Buice et al., 2010; Touboul and Ermentrout, 2011).

The conductance based model introduced in this paper describes the propagation of spikes over the cortical surface and how their effects on post-synaptic responses can be modeled in a channel-specific fashion. In principle—as illustrated in the transfer function analyses—changes in the balance of cortical excitation and inhibition may be modeled more appropriately with conductance based models, relative to classical convolution based models. In particular, these sorts of neural field models characterize the geometry and spatiotemporal dynamics that are supported by intrinsic or lateral interactions on the cortical surface and, implicitly, pharmacological effects on these interactions (such as anaesthetic administration). In the next phase of this work, we will use the conductance based field model described here as an observation or generative model of empirical electrophysiological responses to establish its validity, within the setting of dynamic causal modeling.

### Conflict of interest statement

The authors declare that the research was conducted in the absence of any commercial or financial relationships that could be construed as a potential conflict of interest.
